# Quality Assessment of Minced Poultry Products Including Black Fermented Garlic

**DOI:** 10.3390/foods13010070

**Published:** 2023-12-24

**Authors:** Anna Augustyńska-Prejsnar, Miroslava Kačániová, Małgorzata Ormian, Jadwiga Topczewska, Zofia Sokołowicz, Paweł Hanus

**Affiliations:** 1 Department of Animal Production and Poultry Products Evaluation, Institute of Food Technology and Nutrition, University of Rzeszow, 35-959 Rzeszow, Poland; aaugustynska@ur.edu.pl (A.A.-P.); mormian@ur.edu.pl (M.O.); zsokolowicz@ur.edu.pl (Z.S.); 2 Institute of Horticulture, Faculty of Horticulture and Landscape Engineering, Slovak University of Agriculture, 949 76 Nitra, Slovakia; miroslava.kacaniova@gmail.com; 3 Department of Food Technology and Human Nutrition, Institute of Food and Nutrition Technology, University of Rzeszow, 35-959 Rzeszow, Poland; phanus@ur.edu.pl

**Keywords:** poultry meat, black garlic, microbiological quality, sensory attributes

## Abstract

The aim of this study was to evaluate the effect of the addition of fermented black garlic on the quality of minced poultry products. Treatments were organized in four groups (1%, 2%, 3%, and 4%) containing either black fermented garlic (bg) or fresh garlic (fg), and a control (produced without garlic). The quality assessment of minced poultry products included physicochemical properties (weight losses, pH, colour and shear force), microbiological quality (Enterobacteriaceae, total count of bacteria, lactic acid bacteria, and *Pseudomonas* spp.) and evaluation of sensory attributes. The results showed that the pH values in the black garlic groups, pH 6.06, 6.03, and 6.01, were lower than in the control group, pH 6.16, and tended to decrease during the period of cold storage. As the percentage of black garlic increased, there was a decrease in pH, the value of L* (brightness) from 76.16 in the control group to 48.03 in the group with 4% bg, while the value of b* (yellowing) increased analogously from 12.59 to 16.08. The use of black fermented garlic at 2% as a substitute for fresh garlic is a viable alternative to obtaining product with an acceptable taste and aroma. The addition of 4% black garlic was not acceptable to the assessors.

## 1. Introduction

Black garlic is produced from aging fresh garlic (*Allium sativum* L.) following its fermentation and is one of the best known functional foods. Its fermentation can last between 21 and 72 days at controlled temperature (60–90 °C) and humidity (60–80%) [1,2]. The fermentation of a host of bacteria has been reported to be responsible for fermentation. The fermentation process changes the texture and nutrient content, enhancing its sweetness while boosting its antioxidant properties [3,4,5,6]. Black garlic does not exhibit an unpleasant smell, which is usually associated with fresh garlic. The allicin compound responsible for the typical pungent smell of fresh garlic is converted into water-soluble antioxidants and their compounds, i.e., bioactive alkaloids and flavonoid compounds, including S-allyl-cysteine (SAC) and S-allyl-mercaptocysteine [7,8,9]. Black garlic’s sweet taste is attributed to monosaccharides (glucose and fructose), formed during the breakdown of fructans [10]. Black garlic is distinguished by its dark colour and elastic and malleable jelly-like texture. Its flavour resembles that of dried plum, soy sauce, balsamic vinegar, aniseed and espresso, while perceptible notes of caramel, liquorice and tamarind in the garlic [4] can also be identified. Colour changes occur as a result of a series of non-enzymatic browning reactions between reducing sugars and amino groups of free amino acids [11]. Black garlic after the fermentation process is characterised by a high content of polysaccharides, reducing sugars, proteins, phenolic compounds, organic sulphur compounds, and melanoidins [12]. Furthermore, the Maillard reaction, which occurs during fermentation, increases the concentration of total phenolic acid, flavonoids, 5-hydroxymethylfurfural, melanoidins and thiosulfinates in black garlic [2,3,9,11]. The fermentation process leads to a decreased pH of the garlic as well as decreased moisture content, thus increasing its shelf life [12,13,14]. Fermentation not only affects the basic composition or physicochemical properties of garlic, but also increases the concentration of biologically active compounds [4,15,16,17]. Fermentation promotes the formation of stable compounds with high antioxidant activity, including SAC, a biologically active compound that exhibits antioxidant properties, anticarcinogenic, anti-allergic, anti-diabetic and anti-inflammatory effects [2,18,19,20]. Black garlic has strong antioxidant activity that has been measured using different methods such as reducing power, hydroxy radical scavenging activity and ferrous chelating ability. A study by Ryu and Kang [21] showed that the strong antioxidant properties of black garlic extract can be attributed to its increase in reducing power, hydroxyl radical activity and nitrite uptake. The properties of black garlic make it an attractive additive in meat processing.

Minced meat products are particularly susceptible to oxidation, capable of occurring both during production and storage. Both the processing and the ingredients used can affect the microbiota of the minced meat product and have either a beneficial or negative effect on the quality and safety, e.g., by accelerating the spoilage of the meat product. Due to the development of modern methods for assessing the microbiological quality of meat and meat products using the matrix-assisted laser desorption/ionization (MALDI-TOF MS) method, it has become possible to rapidly assess their quality and identify bacteria content, defined as indicators of hygiene or spoilage [22,23].

The aim of this study was to investigate the effect of the addition of black fermented garlic on the microbiological quality, physicochemical and sensory characteristics of turkey meat balls as an alternative to the addition of fresh garlic commonly used in meat products.

## 2. Materials and Methods

### 2.1. Ingredients of Minced Poultry Products

Poultry meat (36.5 kg of turkey breast muscle) was purchased at the same time prior to the first series of tests at a butcher shop in Rzeszów, Poland. In order to limit raw material variation, the meat was purchased from the same slaughterhouse. The hermetically packed breast fillets were transported in a mobile refrigerator (4 ± 1 °C) to the laboratory. The raw material was then divided into two portions, sealed in vacuum packs (raw weight approximately 0.5 kg) and stored frozen at −20 °C ± 2 °C for 7 (1st series of tests) and 10 days (2nd series of tests). Fermented garlic from the Juleko company (distribution Warsaw, Poland), from organic cultivation (country of origin Spain), purchased from a health food shop, was used as an additive. According to the manufacturer’s declaration, the product contained 100% garlic from EU organic farming certified (No. PL-EKO-07) by the authorized certification body. In addition, heads of fresh organic garlic from BioPlanet were used. The additives used and other ingredients such as natural non-iodised salt and black pepper were purchased from an organic food shop.

### 2.2. Preparation of Minced Poultry Products

Two experimental series were performed. Each series contained thawed meat (0 ± 2 °C), chopped into 4–5 cm cubes and subjected to double grinding in a meat grinder (Zelmer, Rzeszów, Poland) with a mesh of 4 mm hole diameter. The ground raw material was then weighed (Axis laboratory scale, Krakow, Poland) and nine portions (2 kg each) were separated. Nine variants of minced products (poultry meatballs) with a constant recipe composition were prepared (Table 1). The control group consisted of standard minced poultry products in the form of meatballs without the addition of garlic. In the experimental groups, black garlic was added: group 1 (bg)—1%; group 2 (bg)—2%; group 3 (bg)– 3%; group 4 (bg)—4%. Similarly, minced products with fresh garlic of the same proportion were prepared for comparison purposes: group 1 (fg) and group 4 (fg). Both black garlic and fresh garlic had previously been minced in an electric cutter/blender (VBC-32 Hallde, Kista, Sweden) into a paste form. All ingredients were, in each group, put into a mechanical meat mixer with a stainless-steel stirrer (Titanium, Havand, UK) and mixed for 5 min until the ingredients were evenly distributed. Meatballs of 35 ± 2 g were formed from the produced meat masses and subjected to a cooling process in a refrigerated cabinet (FKv36/10 Liebherr, Ulm, Germany) at 4 °C for 0.5 h to fix the shape. Before roasting in the oven, the meatballs were removed from the refrigerated cabinet. An electric oven with steam function (AEG Berlin, Germany), preheated to 180 °C, was used for thermal treatment, in which roasting was carried out until a temperature of 78 ± 2 °C was achieved in the geometrical centre of the product. The temperature was measured using an Amstat digital thermometer (AMT 112 Berlin, Germany) with an external probe. After roasting, the cooked minced meat products were transferred to pans to cool and weighed again to the nearest 0.01 g. Once cooled, the meatballs were packed into separately labelled food storage containers. They were wrapped manually in air-permeable polyethylene food wraps, while maintaining sterility. Microbiological tests, physicochemical properties and sensory evaluation were conducted on day 1 and 3 of storage (4 °C) in a refrigerated cabinet (FK v36/10 Liebherr, Germany).

### 2.3. Assessment of Physicochemical Properties

The weight loss (%) was calculated according to the formula

Weight loss (%) = [(W_1_ − W_2_)/(W_1_)] × 100;

where W_1_ is weight before roasting meatballs, W_2_ is weight after roasting meatballs. Measurements were made in minced poultry products after day 1 and on day 3 of storage in a refrigerated cabinet at 4 °C. The pH of minced poultry products was performed using an HI 99163 pH meter (Hanna Instrument Company, Woonsocket, RI, USA) equipped with an FC2323 electrode (Hanna Instrument Company, Woonsocket, RI, USA). Prior to the measurements, the pH meter was calibrated with buffers pH 4.01 and pH 7.01 (Hanna Instrument Company, Salaj, Romania). Measurements were taken at room temperature (20–22 °C). The average pH value was determined from 6 measurements on the same sample and the procedures were the same for all samples. The external colour and cross-section of minced poultry products were measured using a Konica Minolta Chroma Meter colorimeter (Osaka, Japan), CR—400 head (ø = 11 mm), CIE L*, a* and b* system, D_65_ light source and 2° standard observer. Colour was measured by applying the head of the instrument to the surface that was to be examined. The results were read from the instrument’s display. Ten measurements were taken to calculate the final mean value. This method allowed the evaluation of the colour parameters that describe the brightness of the samples evaluated (parameter L*) and their chromatic colours (parameter a*—red and parameter b*—yellow). Before cutting at sample temperature of 20 °C, the poultry meatballs were formed into 20 × 20 × 50 mm cubes by cutting off the outer surfaces. To measure the cutting force (F max), a Zwick/Roell BT1-FR1.OTH.D14 testing machine (Zwick GmbH & Co. KG., Ulm, Germany) with a Warner-Bratzler single-blade cutting system (one 1.2-mm-thick flat knife with a 60° triangular indentation, the inner edge of which is also the working edge) was used at a head speed of 100 mm-min^−1^ and an initial force of 0.2 N.

### 2.4. Microbiological Analyses

Turkey meatballs (10 g) from all samples tested were used for microbiological conditions, samples were punched using sterile instruments. The samples were punched in a sterile stomacher bag and homogenised for 30 min at 20 °C with 90 mL of water containing 0.1% peptone and a pH of 7.0, Dilutions ranging from 10^−1^ to 10^−3^ were made in series. Microbiological analyses of samples were evaluated on day 1. and 3. Total aerobic microbial counts (cfu/g) were performed using a bacterial counting medium (PCA, Biocorp, Issoire, France). The Petri dishes were incubated for 24 h at 37 °C under aerobic conditions. A medium Pseudomonas Lab-agar with CN supplement (SR0102) (PA, Biocarp, Issoire, France) was used for the isolation of *Pseudomonas* spp. The samples were incubated for 48 h at 25 °C and aerobic conditions. The Enterobacteriaceae family was isolated using Violet red bile glucose agar (VRBL; Biocorp, Issoire, France). A 24 h incubation period at 37 °C was used with the VRBL agar plates. Three duplicate experiments were run [23] for each procedure.

The visible colonies of different appearances obtained in the microbiological status study were identified using MALDI-TOF MS Biotyper analysis. The samples were prepared following the extraction method recommended by the manufacturer (Bruker Daltonik, Bremen, Germany). The bacterial colony was suspended in 900 µL of pure ethanol (Bruker Daltonik, Bremen, Germany) and 300 µL of water (Sigma-Aldrich, St. Louis, MO, USA) and shaken ten times before being centrifuged at 13,000 rpm for two minutes. The pellets were centrifuged multiple times after the supernatant was discarded. Immediately following the removal of the supernatant, 10 µL of 70% formic acid (*v*/*v*) (Bruker Daltonik, Bremen, Germany) was added to the pellets. The mixture was repeatedly centrifuged. Next, it was air-dried at room temperature with 1 µL of the supernatant stained on a polished steel target plate. Each sample received 1 µL of MALDI matrix (saturated HCCA solution, Bruker Daltonik, Bremen, Germany) in 50% acetonitrile and 2.5% trifluoroacetic acid (Sigma-Aldrich, St. Louis, MO, USA). The Microflex LT MALDI-TOF mass spectrometer (Bruker Daltonik, Bremen, Germany), which operates in a linearly positive mode in the mass range of 2000–20,000 Da, automatically created the mass spectacles. The Bruker bacterial standard was used to calibrate the instrument. The MALDI Biotyper 3.0 software (Bruker Daltonik, Bremen, Germany) was utilised to analyse the spectrometric data and obtain the results. The following identification criteria were applied. While a score between 1700 and 1999 indicated probable identification at the genus level, and a score between 2000 and 2299 indicated safe genus identification with potential species identification, the score of between 2300 and 3000 indicated highly probable identification at the species level. In the results, identified microorganisms with a score higher than 2 are used [23].

### 2.5. Sensory Evaluation

The sensory parameter evaluation of refrigerated minced poultry product samples was conducted by a panel of 10 members with at least four years’ experience in performing scaled evaluations. A special-purpose evaluation form was used as part of this study. For proper sensory evaluation, samples of minced poultry products were cut into 2 cm cubes, and were coded and presented to the assessors in white containers. The 5-point hedonic scale was used. The rating attributes were as follows: intensity of aroma (5—very distinct; typical; 4—distinct; typical; 3—weakly distinct; not perceptible; typical; 2—perceptible; 1—not perceptible); desirability of aroma (5—very desirable; typical; 4—desirable; typical; 3—moderately desirable 2—slightly undesirable; 1—very undesirable); taste intensity (5—very desirable; desirable; typical; 4—desirable; typical; 3—slightly desirable; 2—perceptible; atypical; 1—very undesirable); flavour intensity (5—very desirable; typical; 4—desirable; typical; 3—moderately desirable; 2—slightly undesirable; 1—very undesirable); colour of cut section (5—even; desirable; typical; 4—desirable; less even; typical; 3—moderately desirable; not even; altered in places; 2—slightly undesirable; altered in places; 1—very undesirable; altered); texture (5—very desirable; compact; 4—desirable; typical; 3—less desirable; 2—loose or strongly compact 1—separates or very compact); general appearance (5—unobjectionable; desirable; typical; 4—desirable; slightly dry surface; 3—moderately desirable; dry or slightly moist surface; 2—undesirable; slimy; slightly sticky surface; colour locally altered; 1—very undesirable; mucus–sticky surface).

In order to be accurately assessed, samples were coded and presented to the assessors in white containers. The examination was carried out in a suitably prepared room free of foreign smells, at 20 °C and light, eliminating all distracting factors, according to current standards [24]. The panellists did not eat for 2 h prior to the assessment and their mouths were rinsed with water between tests.

### 2.6. Statistical Analysis

The data obtained were tabulated and statistically analysed using Statistica 13.3 [25]. The collected data were checked for normality using the Kolmogorov–Smirnov test with Lilliefors correction. The mean and standard deviation were calculated. The results on the effect of black garlic and fresh garlic addition were verified by one-way analysis of variance. The Tukey test at the 95% confidence level (α = 0.05, α = 0.01) was used to indicate the significance of differences between group means. Differences were considered significant if *p* < 0.05. Results for the effects of black garlic and fresh garlic on the sensory attributes of the minced poultry product were verified using non-parametric Kruskal–Wallis tests.

## 3. Results and Discussion

### 3.1. Evaluation of Physicochemical Characteristics of Minced Poultry Product

The water holding capacity and the binding of fat is an important factor in reducing losses during the heat treatment of meat products [26]. Our study showed that the type of additive used had no effect on the amount of cooking losses (Table 2). Cooking losses were significantly (*p* < 0.05) higher in the control and group 1 compared to that of group 4 and products with 4% additive), when fresh and black fermented garlic were used. The results obtained correspond with the study of Kim et al. [7], where powdered black garlic was added to pork pates, and Jin et al. [27] with the use of minced black garlic in dried pork meatballs.

This study showed that the pH value of minced poultry products decreased (*p* < 0.05) depending on the amount and type of additive used (Table 2). In the garlic-containing products, there was a greater decrease in pH among the group with fermented black garlic. As reported by Cayré et al. [28], the low pH of black garlic of 4.47 may have an impact on pH levels in minced poultry products with its addition. It was observed, in the current study, that the degree of acidification of minced poultry products with black garlic decreased during refrigerated storage, as reflected in Shin et al. [29]. A similar trend was observed in the work of Lishianawati et al. [30], where the degree of acidity of duck meat nuggets decreased with the addition of black garlic powder and also with storage time. Jin et al. [27] demonstrated that with the increasing addition of black garlic in low-salt and low-fat meatballs, the pH value decreased. In addition, a study by Kim et al. [7] reported a decrease in pH with the proportion of added black garlic in pork chops. Barido et al. [26] and Yoon et al. [31] obtained decreases in pH with the addition of black garlic extract similarly in heat-treated chicken breast muscles and pork sausages. According to Huff-Lonergan and Lonergan [32], however, low pH values are unfavourable for the meat industry, as they are a sign of excessive protein denaturation. Meat and meat products with lower pH values have poorer water holding capacity, harder texture, lower yield and perish more quickly.

This study showed that the addition of black garlic had a significant (*p* < 0.05) effect on the colour change in minced poultry products (Table 2). As expected, with the amount of additive used, the colour of the product with black garlic became darker. There was a decrease in the brightness parameter (L*) and an increase in the yellowness (b*) of both the outer colour and the cross-section of the minced meat products. The darkening effect of the products with the inclusion of black garlic is due to its dark colouring. Black garlic is produced during a process similar to caramelisation. It is fermented for more than 30 days at a temperature (60–80 °C) and high humidity, during which it undergoes a Maillard reaction and changes its colour and aroma [27]. Similar results of lowering the brightness parameter (L*) in meat products with the addition of black garlic were obtained by Lishianawati et al. [30], Kim et al. [7], Jin et al. [27] and Mancini et al. [33]. Barido et al. [26], using the addition of black garlic extract to cook chicken breasts, obtained a product with a darker colour relative to the control sample. Moreover, Fernandez-Lopez et al. [34], in a study on the effect of the addition of garlic extract to beef balls, came to similar conclusions. As reported by Barido et al. [35], the addition of black garlic to meat products intensifies yellowing relative to the conventional product. This is confirmed by the results of Mancini et al. [33] and Kim et al. [7], where the addition of black garlic powder increased the value of the b* parameter. According to Shin et al. [29], as the concentration of added black garlic extract increased, the brightness (L*) and redness (a*) decreased and the yellowing (b*) of the pork sausages increased. As part of this study (Table 2), it was shown that there was a brightening of the outer colour and a change in the colour of the cross-section of minced poultry products with 3 days of refrigerated storage. The authors explained this by the presence of antioxidant compounds that delay the formation of metmyoglobin responsible for the darker colour of meat products.

This study showed that the tenderness of minced poultry products measured by cutting force varied according to the amount of additive used. Lower cutting strength was found in products with 4% garlic compared to the other research groups and the control group. A short storage time of 3 days also had no effect on the value of the tenderness parameter shear force value measured by shear force, as confirmed by Lishianawati et al. [30]. In a study by Barido et al. [26], it was reported that the addition of black garlic extracts to cooked chicken breast samples had no effect on the shear force (N) value. In the work of Lishianawati et al. [30], it was also noted that there was no effect of the addition of 1% black garlic on the tenderness of duck meat nuggets. According to Santhi et al. [36], the hardness (tenderness) of meat preparations is most influenced by the type of protein, the quality of fat and the non-meat ingredients used. According to Lund et al. [37], the tenderness of a meat product can change with storage time and is related to protein oxidation, which leads to protein cross-linking and polymerisation. As reported by Barido et al. [38], tenderness is an important attribute that the meat industry seeks to maintain due to its role in ensuring consumer satisfaction. In addition, the texture of meat preparations is primarily shaped by the formulation composition (meat and fat content of the raw material), the degree of protein hydration, and the type and number of additives placed [39]. Differences in shear force values in this group may also be related to lower weight losses.

### 3.2. Microbiological Analyses of Minced Poultry Product

The number of Enterobacteriacea family were not detected in any groups on both days. The total count of bacteria ranged from 1.04 to 1.25 log cfu/g on day 1 and from 1.02 to 1.48 log cfu/g on day 3, while lactic acid bacteria ranged from 1.02 to 1.48 log cfu/g on day 1. and on day 3 were found only in control group, and number of *Pseudomonas* spp. were from 0.98 to 1.54 log cfu/g on 1. day and from 1.12 to 1.82 log cfu/g on 3. day (Table 3). As part of this study, it was found that the use of the addition of black garlic significantly (*p* < 0.05) affected the inhibition of total counts of bacteria and *Pseudomonas*. Samples of minced poultry products with black garlic were more resistant to microbial growth on both the first and third days of storage (Table 3). As such, it can be assumed that the presence of compounds exhibiting antimicrobial properties in the black garlic formulation influenced the inhibition of microbial growth and increased shelf life during storage. The most important antimicrobial compounds contained in black garlic are organosulfur derivatives, i.e., allicin, diallyl sulfide, diallyl disulfide and S-allocysteine [2,18,19]. As reported by Kim et al. [7], the compounds in black garlic have a destructive effect on bacteria (among others, on *Staphylococcus*, *Streptococcus*, *Salmonella*, *Escherichia*, *Klebsiella*, *Proteus*, *Helicobacter*, *Mycobacterium*, and *Clostridium*), protozoa (*Entamoeba histolytica*) and fungi (*Candida albicans*, *Saccharomyces cerevisiae*, *Pichia anomala*, *Hanseniaspora valbyensis*, and *Aspergillus niger*). Of particular importance is allicin, which penetrates into the cell and interacts with cytoplasmic components and enzymes; this compound also blocks lipid and RNA synthesis in bacteria [6,13].

The bioactive effects of black garlic, including antimicrobial activity, are confirmed by the work of Mahros et al. [40] and Javed et al. [41]. Their studies showed that the total count of bacteria and *Pseudomonas* gradually increased in all samples throughout the storage period. However, ground beef with higher levels of garlic showed slower microbial development in the study by Aydin et al. [42]. While being stored at 3 °C, the antimicrobial effects of equivalent concentrations of fresh garlic, garlic powder and garlic oil were examined. It was discovered that the addition of fresh garlic (30 g/kg) or garlic powder (9 g/kg) significantly decreased the aerobic plate count, thereby extending the shelf life of the product [43]. When compared with the control group in the same study, the addition of garlic oil had no discernible impact on the number of microorganisms present [44]. Similar findings were achieved in another investigation, which indicated that garlic oil had only a very modest antibacterial effect [44]. Numerous intrinsic and extrinsic factors can influence microbial growth in meat products; however, the main variables that have a significant impact on bacterial growth are storage temperatures, pH, moisture, oxygen availability, and bacterial traits like endospore [45]. Numerous research has also been conducted on in vitro antimicrobial effects of garlic against particular microorganisms, including bacteria that are multidrug-resistant, and significant inhibition zones have been identified [46,47]. Allicin liquids produced zone diameters of 33 mm when the suggested therapeutic concentration of 500 g/mL (0.0005% wt/vol) was used, and all strains were inhibited at 32 g/mL and killed at 256 g/mL. Cutler and Wilson [48] evaluated the antimicrobial effect of an aqueous extract of allicin (extracted from garlic) on 30 clinical isolates of methicillin-resistant *Staphylococcus aureus*.

**Figure 1:**
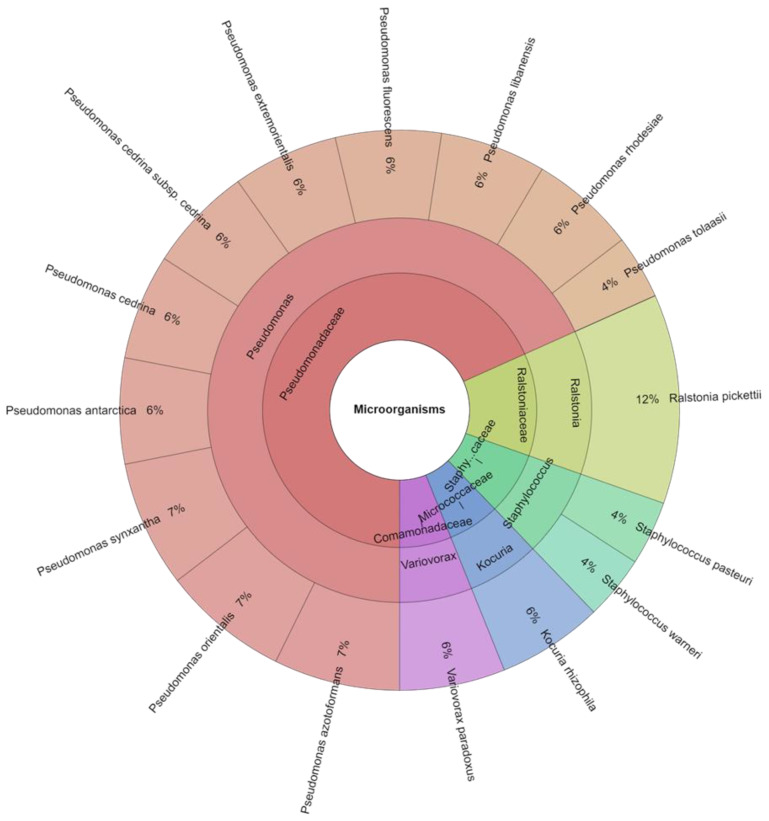
Krona chart of isolated species of microorganisms in 1 day.

**Figure 2:**
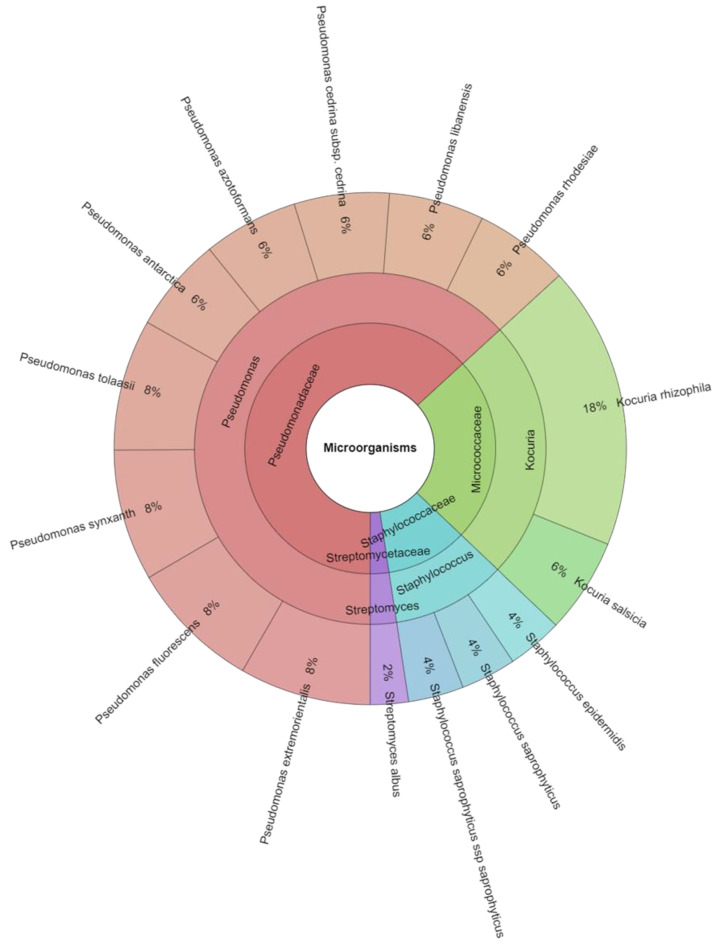
Krona chart of isolated species of microorganisms in 3 day.

A total of 82 isolates were identified from the control and treated groups of meat samples. A total of 5 families, 5 genera, and 16 species were isolated from the groups of samples on the first day (Table 4). The most isolated species in this study were *Palstonia picketii* (12.3%), *Pseudomonas azotoformans* (7.3%), and *Pseudomonas orientalis* (7.3%) followed by *Pseudomonas synxantha* (7.3%). A total of four families, four genera, and 15 species were isolated from the groups of meat after 3 days. The most commonly isolated species was *Kocuria rhizophila* (17.9%), which was added to this group. The other most often isolated species of bacteria from the treated group were *Pseudomonas extremorientalis*, *Pseudomonas fluorescens*, *Pseudomonas synxantha*, and *Pseudomonas tolaasii*—all 8.3%. *Streptococcus*, *Acinetobacter*, and *Pseudomonas* predominated the bacterial community in the thermally treated meatballs at the end of storage, whereas *Pseudomonas*, *Pantoea*, and *Serratia* did so in the control group (Figure 1 and Figure 2). Results from earlier research suggested that variations in the total volatile basic nitrogen (TVB-N) in beef products were related to the growth of *Pseudomonas* spp. during storage [49]. Additionally, it has been demonstrated that *Pseudomonas* spp. have excellent proteinase and amino acid metabolic abilities in meat products [50,51]. The oxygen-limiting conditions found in meat after the rapid expansion and domination of *Pseudomonas* spp. could be the cause of this shift in the bacterial community favouring facultative anaerobes [52].

### 3.3. Sensory Analysis of Minced Poultry Product

Sensory attributes are important from the point of view of consumers who purchase meat products. They are dependent on both the quality of the meat raw material and the additives used [40]. Volatile and non-volatile compounds formed during meat transformation and processing, as well as the proportion of substances enriching the final product, are responsible for shaping the sensory quality determinants of enriched meat products [27]. This study showed that the proportion of black fermented garlic added had a significant effect on the sensory evaluation results obtained (Table 5). It was also shown that in the study group, minced poultry products with 2% black garlic were characterised by the highest desirability of taste and aroma. The minced poultry products with 3% black garlic also received lower marks from the evaluators, but acceptability to the evaluation panel. In terms of both the desirability of aroma, flavour, and colour, products with 4% black garlic were not accepted by the evaluation panel. In the evaluation of the sensory characteristics of minced poultry products, the product with 1% of fresh garlic addition was characterised by the highest desirability of the garlic characteristics evaluated. With the addition of fresh garlic, a reduction in quality characteristics was observed, but products with 3% and 4% addition were not accepted in terms of taste and smell desirability. Lishianawati et al. [30] report that an acceptable level of addition of dried black garlic to duck meat nuggets was 1%. In addition, Kim et al. [7] suggest that a 2% addition of dried black garlic improved the sensory properties (colour, flavour and overall desirability) of pork chops. In a study by Barido et al. [26], the use of black garlic extracts improved meat quality by providing better organoleptic characteristics. In contrast, Shin et al. [29] indicate that the use of 15–22% black garlic extract in the preparation of pork sausages improves sensory attributes.

## 4. Conclusions

This study showed that the addition of black garlic to minced poultry product had an effect on pH, colour, and inhibition of total counts of bacteria and *Pseudomonas*. With increasing levels of fermented black garlic addition, there was a decrease in pH levels as well as the darkening of the colour of the minced poultry product.

Sensory evaluation showed that, in terms of taste and aroma desirability, minced poultry products with a 2% addition of black garlic scored the highest. A higher proportion of 4% black garlic addition was not acceptable to the assessors in terms of colour, flavour and overall desirability. In comparison, minced poultry products with 1% fresh garlic had the highest desirability for sensory attributes.

The results obtained with the use of black garlic as an additive to minced poultry products are promising and worth of further research.

## Figures and Tables

**Table 1:** Composition of minced poultry products (%).

Ingredients	Variants of Product								
	Control Group	Group 1		Group 1		Group 3		Group 4	
		fg	bg	fg	bg	fg	bg	fg	bg
breast muscle meat of turkeys for slaughter	89.30	88.30	88.30	87.30	87.30	86.30	86.30	85.30	85.30
water	10.00	10.00	10.00	10.00	10.00	10.00	10.00	10.00	10.00
black garlic (bg)	0		1.00		2.00		3.00		4.00
fresh garlic (fg)	0	1.00		2.00		3.00		4.00	
salt	0.50	0.50	0.50	0.50	0.50	0.50	0.50	0.50	0.50
pepper	0.20	0.20	0.20	0.20	0.20	0.20	0.20	0.20	0.20

**Table 2:** Effect of the addition of black garlic on the physicochemical characteristics of minced poultry products stored refrigerated (mean and standard deviation).

	Day	Control Group	Addition Share								*p*-Value
			1		2		3		4		
			fg	bg	fg	bg	fg	bg	fg	bg	
Wl (%)	1	14.14 ± 1.21 ^a^	14.99 ± 0.9 9	14.79 ± 1.30 ^a^	13.04 ± 1.12 ^ab^	12.81 ± 0.98 ^ab^	12.58 ± 1.12 ^ab^	12.49 ± 1.12 ^ab^	12.18 ± 1.12 ^b^	12.05 ± 1.12 ^b^	0.0000
pH	1	6.16 ± 0.02 ^a^	6.10 ± 0.02 ^b^	6.05 ± 0.05 ^a^	*6.08 ± 0.04 ^a^	6.03 ± 0.03 ^a^	*6.06 ± 0.03 ^c^	6.01 ± 0.04 ^a^	*6.02 ± 0.04 ^c^	5.98 ± 0.02 ^a^	0.0000
	3	6.21 ± 0.02 ^a^	6.18 ± 0.06 ^b^	6.03 ± 0.04 ^bd^	*6.03 ± 0.07 ^b^	6.04 ± 0.03 ^a^	*6.00 ± 0.03 ^c^	5.97 ± 0.02 ^d^	*5.78 ± 0.05 ^c^	5.92 ± 0.05 ^d^	0.0000
External colour											
L*	1	*76.16 ± 0.94 ^a^	*76.76 ± 1.79 ^a^	63.00 ± 2.79 ^d^	*75.16 ± 2.25 ^ac^	*53.56 ± 0.97 ^e^	*72.40 ± 0.84 ^bc^	*53.07 ± 3.53 ^e^	*73.89 ± 0.58 ^ac^	48.03 ± 1.02 ^f^	0.0000
	3	*78.42 ± 0.96 ^a^	*78.86 ± 0.99 ^a^	63.00 ± 1.61 ^b^	*77.54 ± 1.07 ^a^	*59.29 ± 0.78 ^c^	*76.30 ± 1.09 ^a^	*55.69 ± 1.53 ^d^	*76.87 ± 1.15 ^a^	48.92 ± 2.38 ^e^	0.0000
a*	1	10.05 ± 0.72 ^a^	*7.94 ± 0.84 ^b^	*8.13 ± 0.66 ^b^	*7.22 ± 0.48 ^b^	*8.19 ± 0.28 ^b^	*7.28 ± 0.46 ^b^	7.12 ± 0.83 ^bc^	*7.19 ± 0.14 ^b^	*7.11 ± 0.28 ^c^	0.0000
	3	9.11 ± 0.23 ^a^	*5.62 ± 0.34 ^bd^	*7.20 ± 0.44 ^cf^	*5.16 ± 0.19 ^b^	*6.93 ± 0.25 ^cf^	*5.66 ± 0.42 ^bd^	6.24 ± 0.18 ^e^	*5.40 ± 0.18 ^bd^	*5.91 ± 0.30 ^de^	0.0000
b*	1	12.59 ± 1.27 ^acd^	*12.17 ± 0.74 ^a^	*14.07 ± 1.24 ^ae^	*14.49 ± 1.76 ^ce^	*16.06 ± 0.31 ^be^	*14.81 ± 1.09 ^be^	*15.79 ± 1.09 ^be^	14.59 ± 0.91 ^de^	*16.08 ± 0.76 ^be^	0.0000
	3	10.19 ± 1.19 ^a^	*9.93 ± 0.79 ^a^	*11.80 ± 0.28 ^b^	*11.77 ± 0.65 ^b^	*12.14 ± 1.04 ^b^	*11.22 ± 1.14 ^ab^	*12.57 ± 0.72 ^b^	*11.90 ± 0.36 ^b^	*12.05 ± 0.71 ^b^	0.0000
Colour in cross-section											
L*	1	80.05 ± 1.49 ^a^	79.62 ± 1.34 ^a^	66.06 ± 3.28 ^c^	*81.97 ± 2.67 ^b^	*62.73 ± 1.04 ^d^	*81.23 ± 0.96 ^ab^	*59.42 ± 0.76 ^e^	*81.50 ± 0.98 ^ab^	51.05 ± 1.11 ^f^	0.0000
	3	81.16 ± 0.45 ^a^	77.93 ± 0.76 ^b^	66.50 ± 1.29 ^d^	*79.81 ± 0.75 ^ac^	*57.36 ± 0.43 ^ef^	*80.22 ± 0.48 ^a^	*55.84 ± 1.36 ^f^	*78.13 ± 1.43 ^bc^	50.06 ± 0.47 ^f^	0.0000
a*	1	*8.81 ± 0.44 ^ac^	*8.86 ± 0.48 ^c^	6.85 ± 0.37 ^b^	6.18 ± 0.22 ^b^	6.84 ± 0.82 ^b^	6.13 ± 0.51 ^b^	7.15 ± 0.55 ^b^	*6.74 ± 1.62 ^b^	6.77 ± 0.19 ^b^	0.0000
	3	*7.86 ± 0.35 ^a^	*7.47 ± 0.18 ^a^	6.70 ± 0.13 ^d^	5.52 ± 0.26 ^b^	6.26 ± 0.14 ^e^	5.58 ± 0.14 ^b^	6.58 ± 0.19 ^de^	*4.35 ± 0.39 ^c^	6.83 ± 0.15 ^d^	0.0000
b*	1	7.03 ± 0.12 ^a^	7.61 ± 0.27 ^a^	10.73 ± 0.42 ^c^	7.45 ± 0.36 ^a^	11.63 ± 0.29 ^d^	8.41 ± 0.19 ^b^	*13.23 ± 0.35 ^e^	*8.23 ± 0.22 ^b^	13.98 ± 0.34 ^f^	0.0000
	3	6.27 ± 0.32 ^a^	8.21 ± 0.19 ^b^	10.62 ± 0.36 ^c^	8.04 ± 0.05 ^b^	11.56 ± 0.33 ^e^	10.00 ± 0.48 ^c^	*12.48 ± 0.56 ^d^	*10.70 ± 0.23 ^c^	13.10 ± 0.61 ^d^	0.0000
Sf (N)	1	15.48 ± 1.53 ^a^	16.50 ± 1.88 ^a^	16.56 ± 2.20 ^a^	15.82 ± 1.65 ^a^	15.65 ± 2.30 ^a^	15.62 ± 0.98 ^a^	15.50 ± 2.02 ^a^	13.99 ± 1.96 ^ab^	13.20 ± 1.80 ^b^	0.0000
	3	16.12 ± 2.02 ^a^	16.42 ± 1.60 ^a^	15.02 ± 1.89 ^a^	15.18 ± 1.50 ^a^	14.89 ± 21.93 ^a^	14.98 ± 1.18 ^a^	14.98 ± 1.86 ^a^	13.20 ± 1.89 ^b^	12.86 ± 1.95 ^b^	0.0000

fg—fresh garlic; bg—black garlic; *—marked means differ in columns; ^a–f^—means marked with different letters in the rows differ; Wl—weight loss; Sf—shear force.

**Table 3:** Number of bacteria in turkey meatballs after 3 days (log cfu/g).

Number of Bacteria (log cfu/g)	Day	Control Group	Addition Share							
			Group 1		Group 2		Group 3		Group 4	
			fg	bg	fg	bg	fg	bg	fg	bg
EF	1	nd	nd	nd	nd	nd	nd	nd	nd	nd
	3	nd	nd	nd	nd	nd	nd	nd	nd	nd
TCB	1	1.25 ^a^ ± 0.05	1.15 ^ab^ ± 0.25	1.05 ^b^ ± 0.20	1.15 ^ab^ ± 0.05	nd	1.04 ^b^ ± 0.12	nd	1.05 ^b^ ± 0.08	nd
	3	1.35 ^a^ ± 0.12	1.48 ^a^ ± 0.08	1.15 ^b^ ± 0.15	1.02 ^bc^ ± 0.10	nd	nd	nd	1.30 ^a^ ± 0.20	nd
LAB	1	nd	nd	1.05 ^a^ ± 0.06	1.48 ^b^ ± 0.18	nd	nd	nd	nd	1.02 ^a^ ± 0.20
	3	1.48 ± 0.32	nd	nd	nd	nd	nd	nd	nd	nd
P	1	1.54 ^a^ ± 0.15	1.02 ^b^ ± 0.04	nd	nd	nd	0.98 ^b^ ± 0.08	nd	nd	nd
	3	1.82 ^a^ ± 0.10	1.15 ^b^ ± 0.23	nd	nd	nd	1.12 ^b^ ± 0.15	nd	nd	nd

EF—Enterobacteriacea; TCB—Total count of bacteria; LAB—lactic acid bacteria; P—*Pseudomonas*, fg—fresh garlic; bg—black garlic; nd—not detected; ^a–c^—means marked with different letters in the rows differ.

**Table 4:** Isolated species of bacteria from all groups.

	Control Group	Addition Share								Total
		Group 1		Group 2		Group 3		Group 4		
		fg	bg	fg	bg	fg	bg	fg	bg	
*Kocuria rhizophila*					10	5	5			20
*Kocuria salsicia*			6							6
*Pseudomonas antarctica*	5					5				10
*Pseudomonas azotoformans*						12				12
*Pseudomonas cedrina*						5				5
*Pseudomonas cedrina* subsp. *cedrina*	7					5				12
*Pseudomonas extremorientalis*	7					5				12
*Pseudomonas fluorescens*	7					5				12
*Pseudomonas libanensis*	5					5				10
*Pseudomonas orientalis*	6									6
*Pseudomonas rhodesiae*	6					5				11
*Pseudomonas synxantha*	7					6				13
*Pseudomonas tolaasii*	7					3				10
*Ralstonia pickettii*		2	2		3				3	10
*Staphylococcus epidermidis*									3	3
*Staphylococcus pasteuri*							3			3
*Staphylococcus saprophyticus*		3								3
*Staphylococcus saprophyticus* subsp. *saprophyticus*		3								3
*Staphylococcus warneri*			3							3
*Streptomyces albus*						2				2
*Variovorax paradoxus*	5									5
*Total*										171

**Table 5:** Effect of the addition of black garlic on the sensory quality of stored refrigerated minced poultry products (points).

Parameter	Day	Control Group	Addition Share							
			Group 1		Group 2		Group 3		Group 4	
			fg	bg	fg	bg	fg	bg	fg	bg
Intensity of aroma	1	4.40 ^a^ ± 0.52	4.70 ^a^ ± 0.48	4.20 ^a^ ± 0.63	4.20 ^a^ ± 0.42	4.50 ^a^ ± 0.53	4.40 ^a^ ± 0.52	4.20 ^a^ ± 0.42	3.50 ^b^ ± 0.53	3.40 ^b^ ± 0.52
	3	4.20 ^a^ ± 0.42	4.50 ^a^ ± 0.48	4.00 ^a^ ± 0.42	4.40 ^a^ ± 0.52	4.70 ^a^ ± 0.48	4.00 ^a^ ± 0.47	3.70 ^ab^ ± 0.48	3.40 ^b^ ± 0.52	3.10 ^b^ ± 0.32
Desirability of aroma	1	3.90 ^a^ ± 0.74	4.60 ^b^ ± 0.52	4.00 ^a^ ± 0.47	3.90 ^a^ ± 0.57	4.70 ^b^ ± 0.48	2.50 ^c^ ± 0.53	3.00 ^ab^ ± 0.67	1.80 ^c^ ± 0.63	2.40 ^c^ ± 0.52
	3	3.70 ^b^ ± 0.48	4.25 ^a^ ± 0.27	3.80 ^ab^ ± 0.63	3.80 ^b^ ± 0.42	4.60 ^a^ ± 0.52	2.40 ^c^ ± 0.52	3.00 ^c^ ± 0.42	2.00 ^c^ ± 0.47	2.20 ^c^ ± 0.42
Taste intensity	1	4.00 ^ab^ ± 0.50	4.70 ^a^ ± 0.40	4.40 ^a^ ± 0.42	3.20 ^b^ ± 0.30	4.75 ^a^ ± 0.53	3.00 ^b^ ± 0.52	4.20 ^a^ ± 0.42	1.80 ^c^ ± 0.50	2.40 ^c^ ± 0.40
	3	3,80 ^b^ ± 0.36	4.45 ^a^ ± 0.40	4.20 ^a^ ± 0.40	3,00 ^b^ ± 0.36	4.70 ^a^ ± 0.48	3.00 ^b^ ± 0.40	3.70 ^b^ ± 0.48	1.60 ^c^ ± 0.32	2.10 ^c^ ± 0.51
Flavour intensity	1	3.90 ^b^ ± 0.32	4.60 ^a^ ± 0.52	4.00 ^ab^ ± 0.63	3.70 ^b^ ± 0.48	4.70 ^a^ ± 0.48	2.50 ^c^ ± 0.53	4.40 ^a^ ± 0.32	1.90 ^c^ ± 0.57	2.00 ^c^ ± 0.47
	3	3.90 ^b^ ± 0.74	4.20 ^ab^ ± 0.52	3.90 ^b^ ± 0.30	3.90 ^b^ ± 0.57	4.60 ^a^ ± 0.20	2.50 ^c^ ± 0.53	4.20 ^ab^ ± 0.67	1.80 ^c^ ± 0.63	2.40 ^c^ ± 0.52
Colour of cut section	1	3.80 ^b^ ± 0.30	4.60 ^a^ ± 0.52	4.20 ^ab^ ± 0.40	3.90 ^b^ ± 0.57	3.80 ^b^ ± 0.48	2.50 ^c^ ± 0.53	3.00 ^ac^ ± 0.67	1.65 ^c^ ± 0.60	1.80 ^c^ ± 0.50
	3	3.70 ^b^ ± 0.60	4.40 ^a^ ± 0.32	4.00 ^ab^ ± 0.47	3.80 ^b^ ± 0.50	3.60 ^b^ ± 0.40	2.00 ^c^ ± 0.42	2.80 ^c^ ± 0.40	1.60 ^c^ ± 0.63	1.60 ^c^ ± 0.32
Texture	1	4.40 ^a^ ± 0.52	4.65 ^a^ ± 0.20	4.00 ^ab^ ± 0.47	4.20 ^ab^ ± 0.42	3.80 ^ab^ ± 0.48	4.00 ^ab^ ± 0.40	3.40 ^b^ ± 0.52	1.80 ^c^ ± 0.63	2.40 ^c^ ± 0.52
	3	4.40 ^a^ ± 0.40	4.40 ^a^ ± 0.42	3.80 ^a^ ± 0.32	4.00 ^a^ ± 0.50	3.50 ^ab^ ± 0.53	2.50 ^b^ ± 0.53	3.00 ^ab^ ± 0.67	1.80 ^b^ ± 0.63	2.40 ^b^ ± 0.52
Overall desirability	1	3.90 ^b^ ± 0.60	4.60 ^a^ ± 0.52	4.00 ^ab^ ± 0.47	3.90 ^b^ ± 0.30	4.60 ^a^ ± 0.48	2.40 ^c^ ± 0.50	3.60 ^b^ ± 0.67	1.80 ^d^ ± 0.63	2.00 ^cd^ ± 0.52
	3	3.80 ^b^ ± 0.36	4.40 ^ab^ ± 0.30	3.90 ^b^ ± 0.30	3.80 ^b^ ± 0.50	4.20 ^ab^ ± 0.40	2.50 ^cd^ ± 0.53	3.00 ^c^ ± 0.42	1.80 ^d^ ± 0.63	1.80 ^d^ ± 0.52

^a–d^—means marked with different letters in the rows differ significantly.

## Data Availability

The data that support the findings of this study are available from the corresponding author (J.T.) upon reasonable request. The data are not publicly available due to the implementation of the project of which it is a part.
